# Efficacy of non-invasive brain stimulation for post-stroke depression: a systematic review and meta-analysis

**DOI:** 10.3389/fpsyg.2026.1870615

**Published:** 2026-08-27

**Authors:** Peizhang Huang, Haojie Wang, Cheng Huang, Changjie Mei, Linrong Liao, Yakun Li

**Affiliations:** 1 Department of Rehabilitation Medicine, The First Dongguan Affiliated Hospital, Guangdong Medical University, Guangdong, China; 2 Dongguan Key Laboratory of Intelligent Rehabilitation, Dongguan, Guangdong, China; 3 Dongguan Rehabilitation Medicine Engineering Technology Research Center, Dongguan, Guangdong, China; 4 Department of Rehabilitation Medicine, The Second School of Clinical Medicine, Guangdong Medical University, Dongguan, Guangdong, China; 5 School of Chinese Medicine, Hong Kong Baptist University, Guangdong, China; 6 Department of Neurology, The First Dongguan Affiliated Hospital, Guangdong Medical University, Guangdong, China

**Keywords:** neuromodulation, non-invasive brain stimulation, post-stroke depression, repetitive transcranial magnetic stimulation, transcranial direct current stimulation

## Abstract

**Objective:**

Post-stroke depression (PSD) is a common neuropsychiatric complication after stroke and is associated with poorer functional recovery and quality of life. This systematic review and meta-analysis evaluated the efficacy and safety of non-invasive brain stimulation (NIBS), specifically repetitive transcranial magnetic stimulation (rTMS) and transcranial direct current stimulation (tDCS), for PSD.

**Methods:**

PubMed, Embase, Web of Science, the Cochrane Library, and Scopus were searched from inception to April 2025. Randomized controlled trials comparing rTMS or tDCS with sham stimulation, placebo, usual care, or other control interventions in adults with PSD were eligible. Standardized mean differences (SMDs) with 95% confidence intervals (CIs) were calculated for depressive symptoms and secondary outcomes. Fixed- or random-effects models were selected according to heterogeneity.

**Results:**

Seventeen randomized controlled trials involving 917 participants were included. Compared with control interventions, NIBS was associated with a greater reduction in depressive symptoms (SMD = −1.24, 95% CI: −1.74 to −0.73, *p* < 0.001). Subgroup analyses showed significant effects for both rTMS (SMD = −1.06, 95% CI: −1.59 to −0.52) and tDCS (SMD = −1.94, 95% CI: −3.25 to −0.63), although the difference between modalities was not statistically significant. High-frequency rTMS showed a numerically larger effect than low-frequency rTMS, but the subgroup difference was not significant. NIBS was also associated with improvements in activities of daily living (SMD = 0.80) and quality of life (SMD = 0.83). No serious stimulation-related adverse events were reported.

**Conclusion:**

Current randomized evidence suggests that rTMS and tDCS may improve depressive symptoms in patients with PSD and appear generally well tolerated. However, the certainty of these findings is limited by substantial heterogeneity, small sample sizes in several trials, and variation in stimulation protocols. Further well-designed trials are needed to define optimal stimulation parameters, treatment timing, and long-term clinical effects.

## Introduction

Post-stroke depression (PSD) is among the most frequent neuropsychiatric complications after stroke, affecting approximately one third of survivors in observational cohorts and meta-analyses (Hackett et al., 2005; Mitchell et al., 2017). PSD is clinically important because it is associated with poorer long-term recovery and increased mortality after stroke (Ayerbe et al., 2013; Jørgensen et al., 2016). Despite this burden, diagnosis and treatment remain challenging because PSD is heterogeneous in timing, symptom profile, diagnostic criteria, and outcome assessment (Aben et al., 2001; De Ryck et al., 2014). Selective serotonin reuptake inhibitors and other antidepressants remain common treatment options, but their use may be limited by variable efficacy, bleeding concerns, drug interactions, and adherence problems (Allida et al., 2020; Hackam and Mrkobrada, 2012; Robinson et al., 2008). These limitations have encouraged investigation of non-pharmacological interventions that can be integrated with post-stroke rehabilitation.

Non-invasive brain stimulation (NIBS) provides a biologically plausible approach for PSD by modulating cortical excitability and mood-related fronto-limbic networks. The present review focused on repetitive transcranial magnetic stimulation (rTMS) and transcranial direct current stimulation (tDCS) because these two modalities have the largest randomized evidence base in PSD and were the techniques prespecified in our eligibility criteria (Bucur and Papagno, 2018; Lefaucheur et al., 2014). Both approaches have most commonly targeted the dorsolateral prefrontal cortex (DLPFC), a region implicated in affective control and post-stroke network reorganization (Bucur and Papagno, 2018; Lefaucheur et al., 2014). Other stimulation protocols and modalities, including theta-burst protocols and transcranial alternating current stimulation, are emerging in depression research (Alexander et al., 2019; Zhang et al., 2024); however, the currently available PSD evidence for these modalities remains less extensive and was therefore outside the scope of this review.

Evidence for tDCS has also expanded beyond clinic-based protocols. Home-based tDCS has recently been evaluated as a potentially scalable approach for depressive disorders, and a recent systematic review and meta-analysis in Scientific Reports suggested that supervised or home-administered tDCS may reduce depressive and anxiety symptoms while requiring careful attention to adherence and adverse-event monitoring (Moshfeghinia et al., 2025). Although these findings are not specific to PSD, they highlight the feasibility advantages of electrical stimulation and the importance of considering delivery setting when interpreting the clinical applicability of tDCS. In PSD, however, patients often have neurological deficits, cognitive impairment, or functional limitations that may require more structured supervision than populations with primary depressive disorders.

Several reviews have summarized NIBS for PSD, but important questions remain unresolved, including the relative effects of rTMS and tDCS, whether stimulation frequency modifies rTMS efficacy, the consistency of effects on secondary rehabilitation outcomes, and the safety profile across randomized trials (Bucur and Papagno, 2018; Shen et al., 2017; Wang et al., 2025). Therefore, we conducted an updated systematic review and meta-analysis of randomized controlled trials to evaluate the efficacy and safety of rTMS and tDCS for PSD. We also examined modality-specific and frequency-specific effects and summarized effects on activities of daily living and quality of life to clarify the potential clinical role of NIBS within post-stroke rehabilitation.

## Methods

### Study design and registration

This meta-analysis was performed in accordance with the Cochrane Handbook for Systematic Reviews of Interventions (Cumpston et al., 2019) and reported following the PRISMA statement. The review protocol was registered in the International Prospective Register of Systematic Reviews (PROSPERO; CRD420251072719).

#### Search strategy

A comprehensive search was conducted in PubMed, Embase, Web of Science, the Cochrane Library, and Scopus from database inception to April 2025. The search combined controlled vocabulary terms (e.g., MeSH or Emtree terms where available) and free-text terms related to stroke, post-stroke depression, non-invasive brain stimulation, rTMS, and tDCS. Terms were searched in title/abstract and keyword fields where supported by the database. Boolean operators (AND/OR) were used to combine concepts and maximize sensitivity. Reference lists of included studies and relevant reviews were manually screened; no additional eligible trials were identified through this process. All records were imported into EndNote X9 for deduplication. A detailed database-specific search strategy is provided in Supplementary Table S1.

#### Eligibility criteria

Study selection followed the PICOS framework (Amir-Behghadami and Janati, 2020). Eligible studies were randomized controlled trials assessing rTMS or tDCS in adults diagnosed with PSD. Interventions could be delivered alone or in combination with pharmacological, behavioral, or rehabilitation therapies. Eligible comparators included sham stimulation, placebo interventions, usual care, antidepressant medication, or other control conditions. Depression severity was assessed using validated rating scales, including but not limited to the Hamilton Depression Rating Scale, Beck Depression Inventory, Patient Health Questionnaire-9, or Self-Rating Depression Scale. Secondary outcomes included activities of daily living, functional independence, quality of life, and stroke-related recovery measures when reported. Studies were excluded if they were not randomized, lacked a control group, did not enroll a stroke or PSD population, or did not provide sufficient data for quantitative synthesis.

#### Study selection and data extraction

Two reviewers (P. H. and H. W.) independently screened titles and abstracts and then assessed the full texts of potentially eligible articles. Disagreements were resolved through discussion, and a third reviewer (L. L.) adjudicated when consensus could not be reached. Data were extracted using a predefined form that captured author, year, country, sample size, participant characteristics, time since stroke, baseline depression severity, study design, stimulation modality, stimulation parameters, comparator condition, outcomes, and adverse events. When multiple depression scales were reported, clinician-rated instruments were prioritized over self-report measures when possible.

#### Quality assessment

The risk of bias of each included study was evaluated using the revised Cochrane Risk of Bias tool for randomized trials (RoB 2.0) (Sterne et al., 2019). The assessment covered five domains: the randomization process, deviations from intended interventions, missing outcome data, measurement of the outcome, and selection of the reported result. Two reviewers independently rated each domain as low risk, some concerns, or high risk, and disagreements were resolved by discussion.

#### Data synthesis and statistical analysis

All statistical analyses were conducted using Review Manager software (RevMan, version 5.4; Cochrane Collaboration) and R software (version 4.2.0; R Foundation for Statistical Computing, Vienna, Austria). Continuous outcomes were pooled as standardized mean differences (SMDs) with 95% CIs because different depression, functional, and quality-of-life scales were used across studies. Between-study heterogeneity was assessed using Cochran’s Q test and the *I*^2^ statistic. A fixed-effect model was used when heterogeneity was low (*I*^2^ < 50%); otherwise, a random-effects model was applied. Prespecified subgroup analyses were conducted according to NIBS type (rTMS vs. tDCS), rTMS frequency (high vs. low frequency), post-stroke stage, and concurrent pharmacological therapy when data were available. Sensitivity analyses were performed by sequentially omitting individual studies. Publication bias was assessed visually using funnel plots and statistically using Egger’s regression test, with *p* < 0.10 indicating potential small-study effects.

## Results

### Study search results

**Figure 1:**
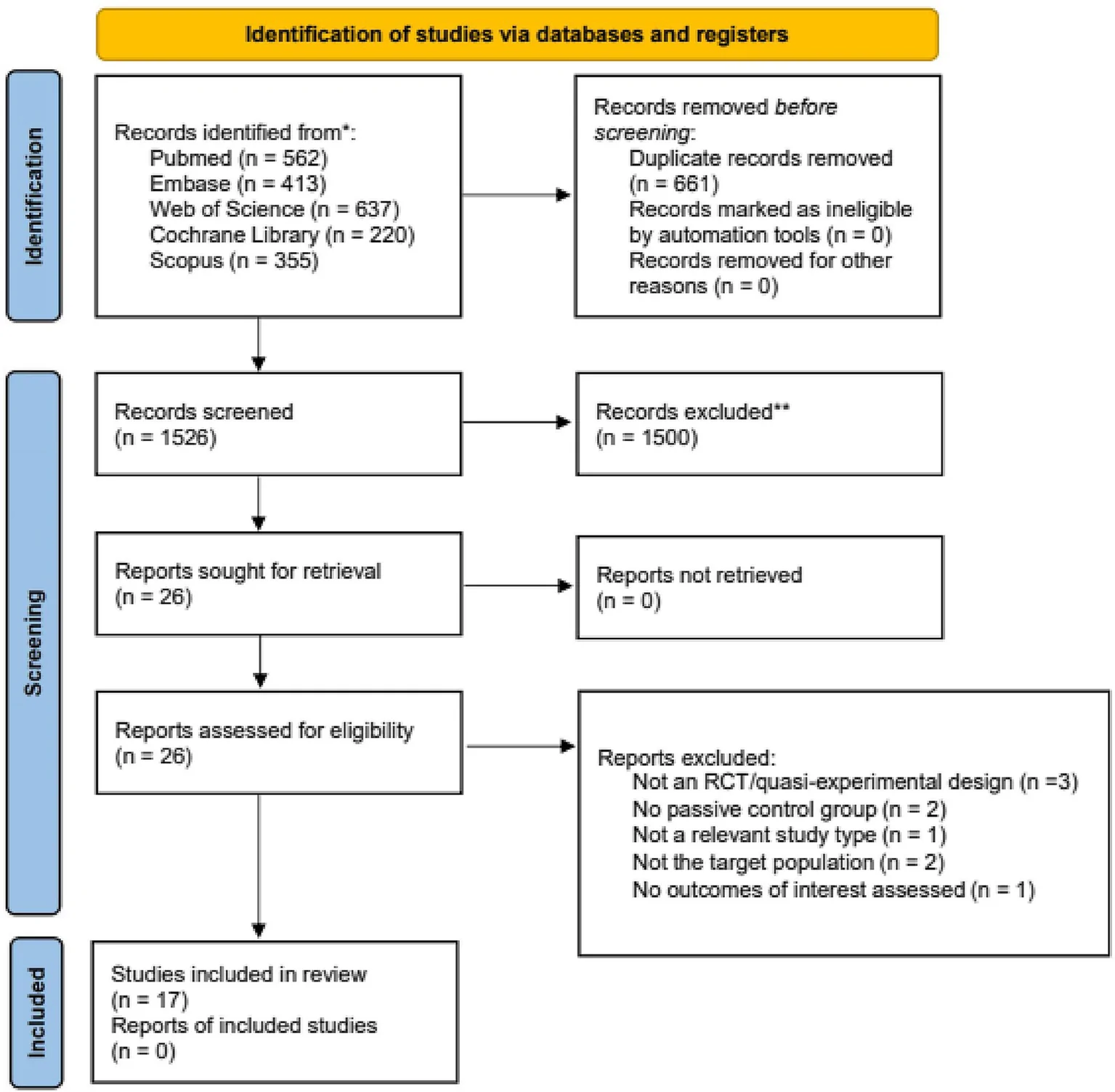
PRISMA flow diagram of study selection process.

A total of 2,187 records were identified across five electronic databases: PubMed (*n* = 562), Embase (*n* = 413), Web of Science (*n* = 637), the Cochrane Library (*n* = 220), and Scopus (*n* = 355). Manual screening of reference lists did not identify additional eligible records. After removal of 661 duplicates, 1,526 unique records were screened by title and abstract, of which 1,500 were excluded. Twenty-six full-text articles were assessed for eligibility, and nine were excluded because of non-randomized or quasi-experimental design (*n* = 3), absence of an eligible control group (*n* = 2), study type inconsistent with the review purpose (*n* = 1), population not matching the inclusion criteria (*n* = 2), or lack of depression-related outcomes (*n* = 1). Ultimately, 17 randomized controlled trials (An et al., 2017; Duan et al., 2023; Gu and Chang, 2017; Hordacre et al., 2021; Hu et al., 2024; Jorge et al., 2004; Kazinczi et al., 2025; Kim et al., 2017; Kim et al., 2010; Li et al., 2024; Ross et al., 2023; Valiengo et al., 2017; Xu et al., 2024; Yu and He, 2021; Zanão et al., 2025; Zhang et al., 2020; Zhu et al., 2022) were included in the final analysis (Figure 1).

#### Risk of bias

**Figure 2:**
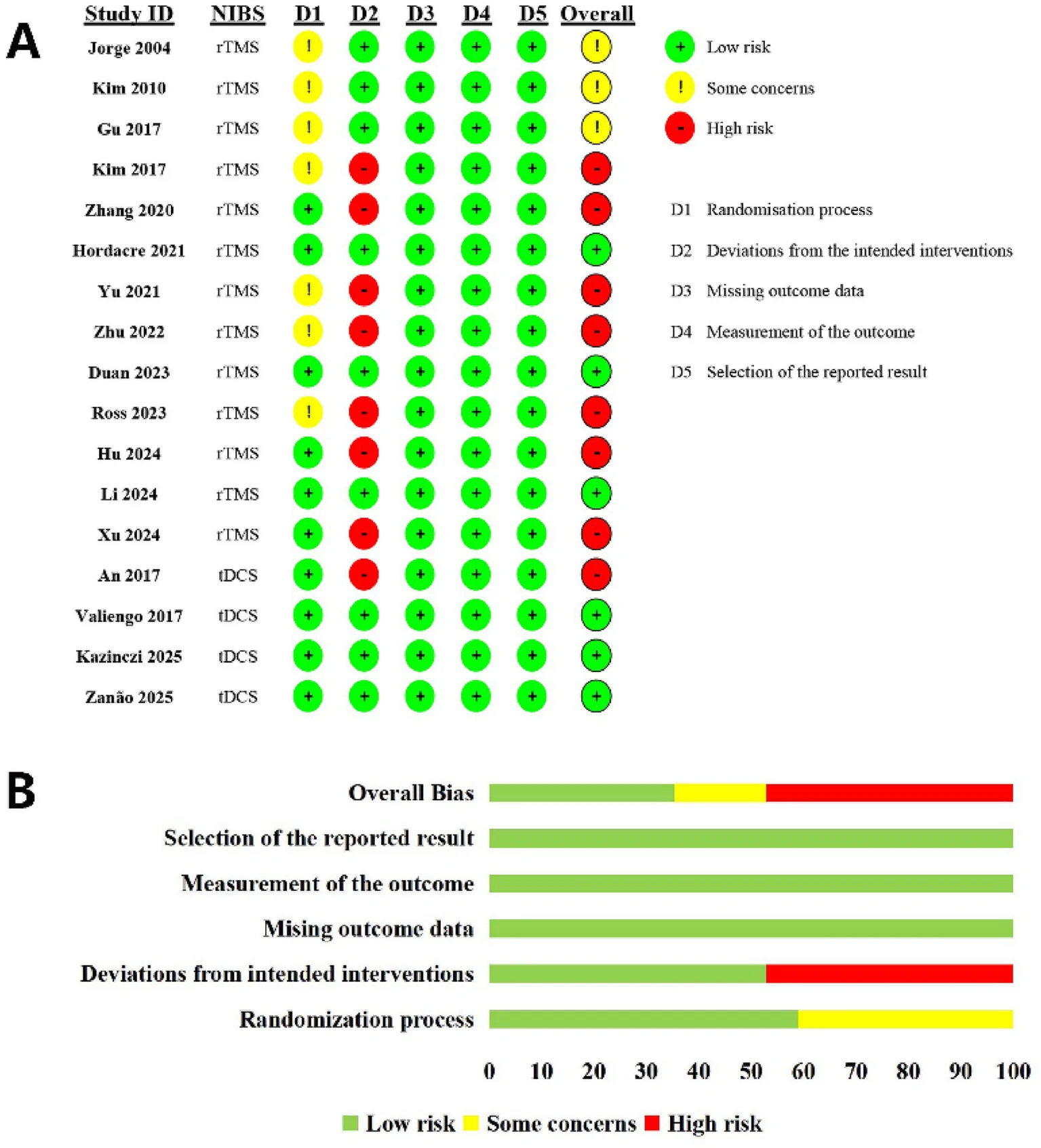
Risk-of-bias assessment of included studies. **(A)** Risk-of-bias judgments for each study across five domains. **(B)** Summary distribution of risk-of-bias judgments across all domains and studies.

An overview of the risk-of-bias assessment is shown in Figure 2. The domain with the highest proportion of high-risk judgments was deviations from intended interventions, with approximately 35% of trials classified as high risk. The randomization process also showed methodological limitations, with around 25% of trials judged as high risk and an additional 35% raising some concerns, mainly because of insufficient reporting of sequence generation or allocation concealment. In contrast, missing outcome data, outcome measurement, and selection of the reported result were generally rated more favorably, with more than 85% of studies considered low risk in these domains and no study judged as high risk. Overall, approximately 35% of included trials were rated as high risk, 18% as having some concerns, and 47% as low risk.

### Characteristics of included studies

A total of 17 randomized controlled trials involving 917 patients with PSD were included in the quantitative synthesis (Table 1). The studies were conducted in China, Korea, the United States, Brazil, Hungary, and Australia. Sample sizes ranged from 10 to 113 participants, with mean ages ranging from approximately 48–68 years. Thirteen study arms evaluated rTMS, and four evaluated tDCS. Most trials used sham stimulation as the comparator, while some used usual care or adjunctive pharmacological or behavioral therapy. rTMS protocols typically targeted the left DLPFC and used frequencies ranging from 1 to 10 Hz, intensities of 80–120% of the resting motor threshold, and treatment durations of 2–8 weeks. tDCS protocols generally delivered 2 mA for 12–30 min per session. Depression was assessed using validated scales such as the HAMD/HDRS, BDI, SDS, or PHQ-9. Several trials also reported secondary outcomes, including activities of daily living, functional independence, or quality of life.

**Table 1:** Characteristics of included randomized controlled trials investigating the effects of non-invasive brain stimulation on post-stroke depression.

Author, year	Country	Study design	Intervention	Onset time	Sample size (M/F)	Age, mean ± SD/ SE (y)	Baseline depression score, mean ± SD/SE	Stroke duration, mean ± SD/SE	Electrode location	NIBS intervention	Outcomes
Jorge et al. (2004)	USA	RCT	EG: rTMS CG: sham rTMS	Chronic	EG: 10 (6/4) CG: 10 (5/5)	EG: 63.1 ± 8.1 CG: 66.5 ± 12.2	EG: 20.1 ± 6.7 (HAMD-17) CG: 20.8 ± 6.0 (HAMD-17)	NR	Left-DLPFC	rTMS, frequency: 10 Hz; intensity: 110% RMT; delivery: 20 trains of 5 s duration; 5 sessions/w, 2 w	Depression (HAMD-17)
Kim et al. (2010)	Korea	RCT	EG: rTMS (10 Hz) CG: sham rTMS	Chronic	EG: 6 (4/2) CG: 6 (4/2)	EG: 53.5 ± 16.9 CG: 66.8 ± 17.2	EG: 12.7 ± 9.9 (BDI) CG: 18.1 ± 8.8 (BDI)	EG: 241.2 ± 42.5 (d) CG: 69.7 ± 39.0 (d)	Left-DLPFC	rTMS, frequency: 10 Hz; intensity: 80% RMT; delivery: a total of 450 pulses, 15 trains of 1 s duration with 10 s ITI; 5 sessions/w, 2 w	Depression (BDI), ADL (MBI)
Kim et al. (2010)	Korea	RCT	EG: rTMS (1 Hz) CG: sham rTMS	Chronic	EG: 6 (2/4) CG: 6 (4/2)	EG: 68.3 ± 7.4 CG: 66.8 ± 17.2	EG: 14.5 ± 11.0 (BDI) CG: 18.1 ± 8.8 (BDI)	EG: 404.4 ± 71.7 (d) CG: 69.7 ± 39.0 (d)	Left-DLPFC	rTMS, frequency: 1 Hz; intensity: 80% RMT; delivery: a total of 900 pulses, 3 trains of 5 min duration with 1 min ITI; 5 sessions/w, 2 w	Depression (BDI), ADL (MBI)
An et al. (2017)	Korea	RCT	EG: tDCS CG: sham tDCS	Chronic	EG: 20 (17/3) CG: 20 (13/7)	EG: 51.0 ± 11.7 CG: 62.4 ± 12.4	EG: 38.8 ± 4.7 (BDI) CG: 39.0 ± 4.6 (BDI)	EG: 14.6 ± 6.3 (m) CG: 14.7 ± 5.9 (m)	Left-DLPFC	tDCS, intensity: 2 mA; session duration: 30 min; 5 sessions/w, 4 w	Depression (BDI), QOL (SS-QOL)
Gu and Chang (2017)	Korea	RCT	EG: rTMS CG: sham rTMS	Chronic	EG: 12 (6/6) CG: 12 (5/7)	EG: 58.1 ± 8.7 CG: 58.3 ± 7.8	EG: 22.9 ± 5.6 (BDI) CG: 22.2 ± 4.1 (BDI)	EG: 10.3 ± 2.7 (m) CG: 10.1 ± 2.3 (m)	Left-DLPFC	rTMS, frequency: 10 Hz; intensity: 110% RMT; delivery: a total of 1,000 pulses, 1 sequence in 5 s with 60 s ITI; 20 sequences/d, 5 sessions/w, 2 w	Depression (BDI)
Kim et al. (2017)	Korea	RCT	EG: rTMS CG: sham rTMS	Chronic	EG: 22 (18/4) CG: 22 (13/9)	EG: 52.6 ± 10.6 CG: 64.3 ± 11.5	EG: 23.0 ± 7.9 (BDI) CG: 24.0 ± 11.6 (BDI)	NR	P3 and P4	rTMS, frequency: 5 Hz; intensity: 90% RMT; delivery: 20 trains of 5 s duration with 55 s ITI; 5 sessions/w, 4 w	Depression (BDI), ADL (FIM)
Valiengo et al. (2017)	Brazil	RCT	EG: tDCS CG: sham tDCS	Chronic	EG: 24 (12/12) CG: 24 (12/12)	EG: 62.2 ± 12.3 CG: 61.3 ± 10.6	EG: 22.6 ± 3.2 (HDRS-17) CG: 21.1 ± 3.5 (HDRS-17)	EG: 11.1 ± 2 (m) CG: 17.4 ± 3.3 (m)	Left-DLPFC	tDCS, intensity: 2 mA; session duration: 30 min; 12 sessions, 6 w	Depression (HDRS-17)
Zhang et al. (2020)	China	RCT	EG: rTMS plus nicergoline CG: nicergoline	NR	EG: 57 (36/21) CG: 56 (33/23)	EG: 63.67 ± 6.04 CG: 63.28 ± 5.91	EG: 27.87 ± 2.34 (HAMD) CG: 28.06 ± 2.71 (HAMD)	NR	Left-DLPFC	rTMS, frequency: 10 Hz; intensity: 80% RMT; session duration: 20 min; 5 sessions/w, 4 w	Depression (HAMD), QOL (WHOQOL-100)
Hordacre et al. (2021)	Australia	RCT	EG: rTMS CG: sham rTMS	Chronic	EG: 6 (5/1) CG: 5 (4/1)	EG: 63.3 ± 11.0 CG: 61.6 ± 12.4	EG: 23.0 ± 7.9 (BDI) CG: 24.0 ± 11.6 (BDI)	EG: 5.5 ± 3.3 (y) CG: 3.9 ± 3.0 (y)	Left-DLPFC	rTMS, frequency: 10 Hz; intensity: 110% RMT; delivery: a total of 3,000 pulses, 40 stimulations during 4 s with 26 s ITI; session duration: 37.5 min; 5 sessions/w, 2 w	Depression (BDI)
Yu and He (2021)	China	RCT	EG: rTMS plus fluoxetine CG: fluoxetine	Acute	EG: 60 CG: 55	EG: 55.91 ± 8.76 CG: 55.75 ± 9.02	EG: 64.0 ± 4.0 (SDS) CG: 64.5 ± 4.8 (SDS)	NR	Left-DLPFC	rTMS, frequency: 10 Hz; intensity: 90% RMT; delivery: 1 sequence in 4 s; 20 sequences/d, 3 sessions/w, 8 w	Depression (SDS), QOL (SF-36)
Zhu et al. (2022)	China	RCT	EG: rTMS plus citalopram CG: sham rTMS plus citalopram	Chronic	EG: 50 (27/23) CG: 50 (24/26)	EG: 48.25 ± 12.82 CG: 48.13 ± 13.42	EG: 24.40 ± 3.01 (HAMD-17) CG: 24.54 ± 2.27 (HAMD-17)	EG: 4.15 ± 1.82 (y) CG: 4.23 ± 2.25 (y)	Left-DLPFC	rTMS, frequency: 5 Hz; intensity: 60% RMT; delivery: a total of 800 pulses, 4 stimulations during 4 s with 56 s ITI; session duration: 20 min; 5 sessions/w, 8 w	Depression (HAMD-17)
Duan et al. (2023)	China	RCT	EG: rTMS plus MBSR CG: sham rTMS plus MBSR	Chronic	EG: 23 (19/4) CG: 24 (20/4)	EG: 58.30 ± 13.06 CG: 53.63 ± 13.01	EG: 19.04 ± 2.16 (HAMD-17) CG: 19.13 ± 3.05 (HAMD-17)	EG: 72.57 ± 20.62 (d) CG: 74.00 ± 18.55 (d)	Left-DLPFC	rTMS, frequency: 10 Hz; intensity: 80% RMT; delivery: a total of 1,400 pulses, 70 stimulations during 4 s with 56 s ITI; session duration: 20 min; 5 sessions/w, 4 w	Depression (HAMD-17), ADL (MBI)
Ross et al. (2023)	USA	RCT	EG: rTMS plus AEx CG: AEx	Chronic	EG: 6 (3/3) CG: 4 (3/1)	EG: 62.3 ± 12.5 CG: 51.5 ± 18.4	EG: 15.3 ± 4.9 (PHQ-9) CG: 12.3 ± 1.7 (PHQ-9)	NR	Left-DLPFC	rTMS, frequency: 10 Hz; intensity: 120% RMT; delivery: 5000 pulses/per session; 3 sessions/w, 8 w	Depression (PHQ-9), QOL (SIS)
Hu et al. (2024)	China	RCT	EG: rTMS plus levetiracetam CG: levetiracetam	NR	EG: 53 (31/22) CG: 68 (34/34)	EG: 67.68 ± 13.48 CG: 64.56 ± 12.81	EG: 25.15 ± 6.63 (HAMD) CG: 24.35 ± 6.81 (HAMD)	NR	M1	rTMS, frequency: 0.5 Hz; intensity: 90% RMT; delivery: a total of 1,200 during 40 min; 1 w	Depression (HAMD), QOL (GQOL-74)
Li et al. (2024)	China	RCT	EG: rTMS CG: sham rTMS	Chronic	EG: 15 (10/5) CG: 15 (8/7)	EG: 63.67 ± 13.89 CG: 56.60 ± 13.70	EG: 15.47 ± 5.45 (HDRS-17) CG: 16.60 ± 5.38 (HDRS-17)	EG: 31.07 ± 19.96 (d) CG: 28.80 ± 19.85 (d)	Left-DLPFC	rTMS, frequency: 10 Hz; intensity: 100% RMT; delivery: a total of 1,170 pulses, 30 stimulations during 30 s with 28 s ITI; 5 sessions/w, 4 w	Depression (HDRS-17)
Xu et al. (2024), (10 Hz)	China	RCT	EG: rTMS (10 Hz) CG: sham rTMS	NR	EG: 25 (14/11) CG: 23 (11/12)	EG: 60.62 ± 11.01 CG: 59.6 ± 10.54	EG: 28.72 ± 7.87 (HAMD-17) CG: 29.87 ± 5.82 (HAMD-17)	NR	Left-DLPFC	rTMS, frequency: 10 Hz; intensity: 90% RMT; delivery: a total of 3,000 pulses, 75 stimulations during 4 s with 26 s ITI; 5 sessions/w, 2 w	Depression (HAMD-17), ADL (MBI)
Xu et al. (2024), (1 Hz)	China	RCT	EG: rTMS (1 Hz) CG: sham rTMS	NR	EG: 26 (14/12) CG: 23 (11/12)	EG: 58.15 ± 11.28 CG: 59.6 ± 10.54	EG: 29.35 ± 6.72 (HAMD-17) CG: 29.87 ± 5.82 (HAMD-17)	NR	Left-DLPFC	rTMS, frequency: 1 Hz; intensity: 90% RMT; delivery: a total of 1800 pulses, 1 stimulations during 1 s with 0 s ITI; 5 sessions/w, 2 w	Depression (HAMD-17), ADL (MBI)
Kazinczi et al. (2025)	Hungary	RCT	EG: tDCS plus ICCT CG: sham tDCS plus ICCT	Chronic	EG: 11 (6/5) CG: 14 (11/3)	EG: 54.27 ± 12.97 CG: 61.54 ± 9.36	EG: 17.36 ± 8.95 (BDI) CG: 9.21 ± 6.53 (BDI)	NR	Fpz, Fz, Cz, Pz, and Oz	tDCS, intensity: 2 mA; session duration: 12 min; 10 d	Depression (BDI)
Zanão et al. (2025)	Brazil	RCT	EG: tDCS CG: sham tDCS	Chronic	EG: 24 (12/12) CG: 24 (12/12)	EG: 62.2 ± 12.3 CG: 61.3 ± 10.8	EG: 22.8 ± 3.14 (HDRS-17) CG: 21.2 ± 3.57 (HDRS-17)	NR	Left-DLPFC	tDCS, intensity: 2 mA; session duration: 30 min; 18 sessions, 6 w	Depression (HDRS-17)

NIBS, non-invasive brain stimulation; EG, experimental group; CG, control group; RCT, randomized controlled trial; tDCS, transcranial direct current stimulation; rTMS, repetitive transcranial magnetic stimulation; DLPFC, dorsolateral prefrontal cortex; BDI, Beck Depression Inventory; QOL, quality of life; ADL, activities of daily living; SSQOL, Stroke-Specific Quality of Life; SF-36, Short Form 36 Health Survey; HDRS-17, 17-item Hamilton Depression Rating Scale; ICCT, inhibitory control training; HAMD-17, 17-item Hamilton Depression Rating Scale; SDS, Self-Rating Depression Scale; MBI, Modified Barthel Index; WHOQOL-100, World Health Organization Quality of Life-100; GQOL-74, Generic Quality of Life Inventory-74; M1, primary motor cortex; RMT, resting motor threshold; ITI, intertrain interval; AEx, aerobic exercise; PHQ-9, Patient Health Questionnaire-9; SIS, Stroke Impact Scale; MBSR, mindfulness-based stress reduction; FIM, Functional Independence Measure; NR, not reported.

#### Therapeutic impact of NIBS on depression

**Figure 3:**
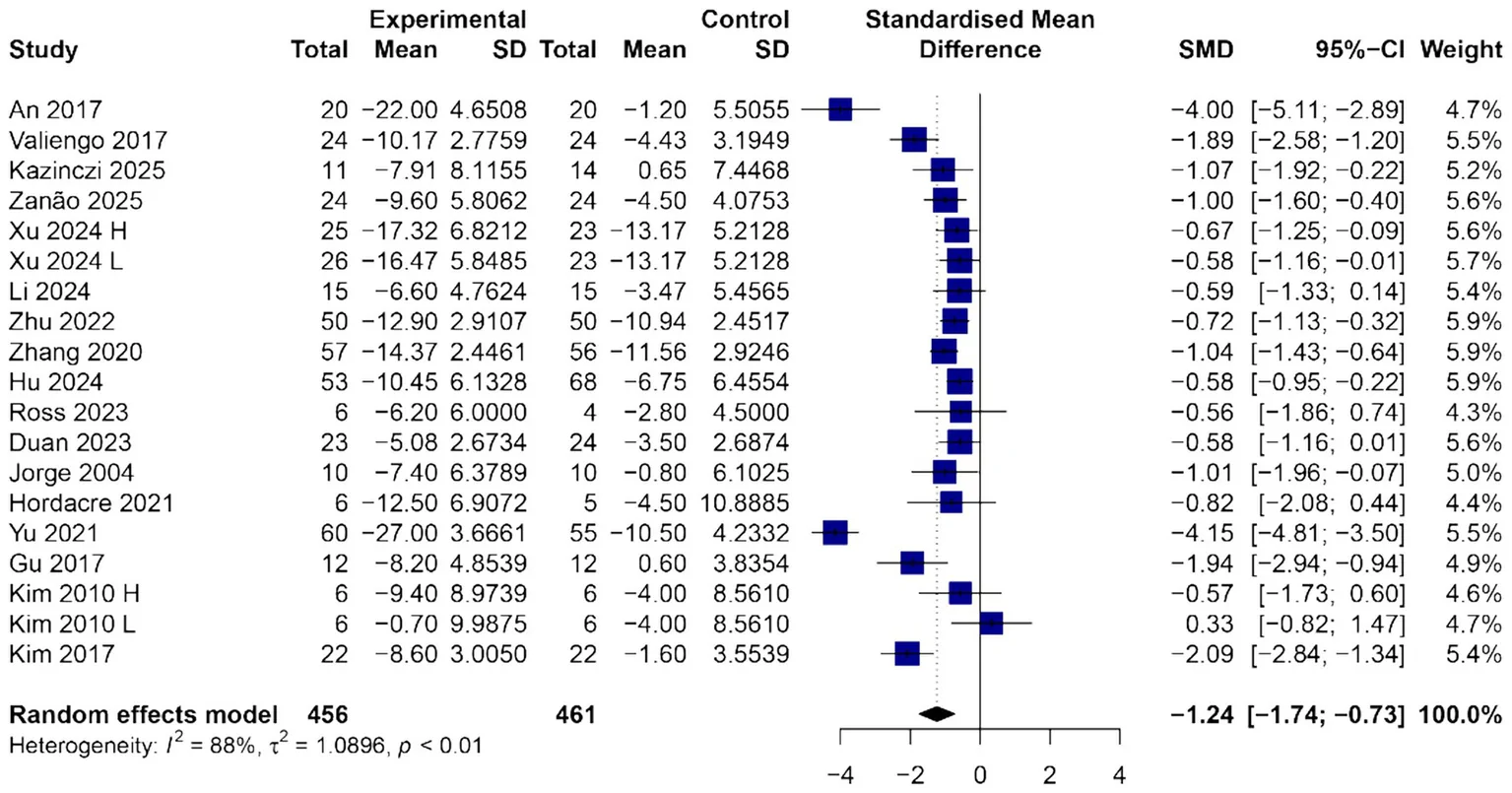
Forest plot of the overall effect of NIBS on depressive symptoms in patients with PSD.

Seventeen studies (An et al., 2017; Duan et al., 2023; Gu and Chang, 2017; Hordacre et al., 2021; Hu et al., 2024; Jorge et al., 2004; Kazinczi et al., 2025; Kim et al., 2017; Kim et al., 2010; Li et al., 2024; Ross et al., 2023; Valiengo et al., 2017; Xu et al., 2024; Yu and He, 2021; Zanão et al., 2025; Zhang et al., 2020; Zhu et al., 2022) involving 917 participants were included in the depression meta-analysis, with 456 allocated to NIBS interventions and 461 to control conditions (Figure 3). The pooled random-effects model showed that NIBS significantly reduced depressive symptoms compared with control interventions (SMD = −1.24, 95% CI: −1.74 to −0.73), corresponding to a moderate-to-large effect. Heterogeneity was substantial (*I*^2^ = 88%, τ^2^ = 1.0896, *p* < 0.01), indicating considerable variation in treatment effects across trials. The largest effect was reported in one tDCS trial (Valiengo et al., 2017), whereas one rTMS study (Kim et al., 2010) showed a small, non-significant effect. Study weights were broadly similar, suggesting that no single trial dominated the pooled estimate.

**Figure 4:**
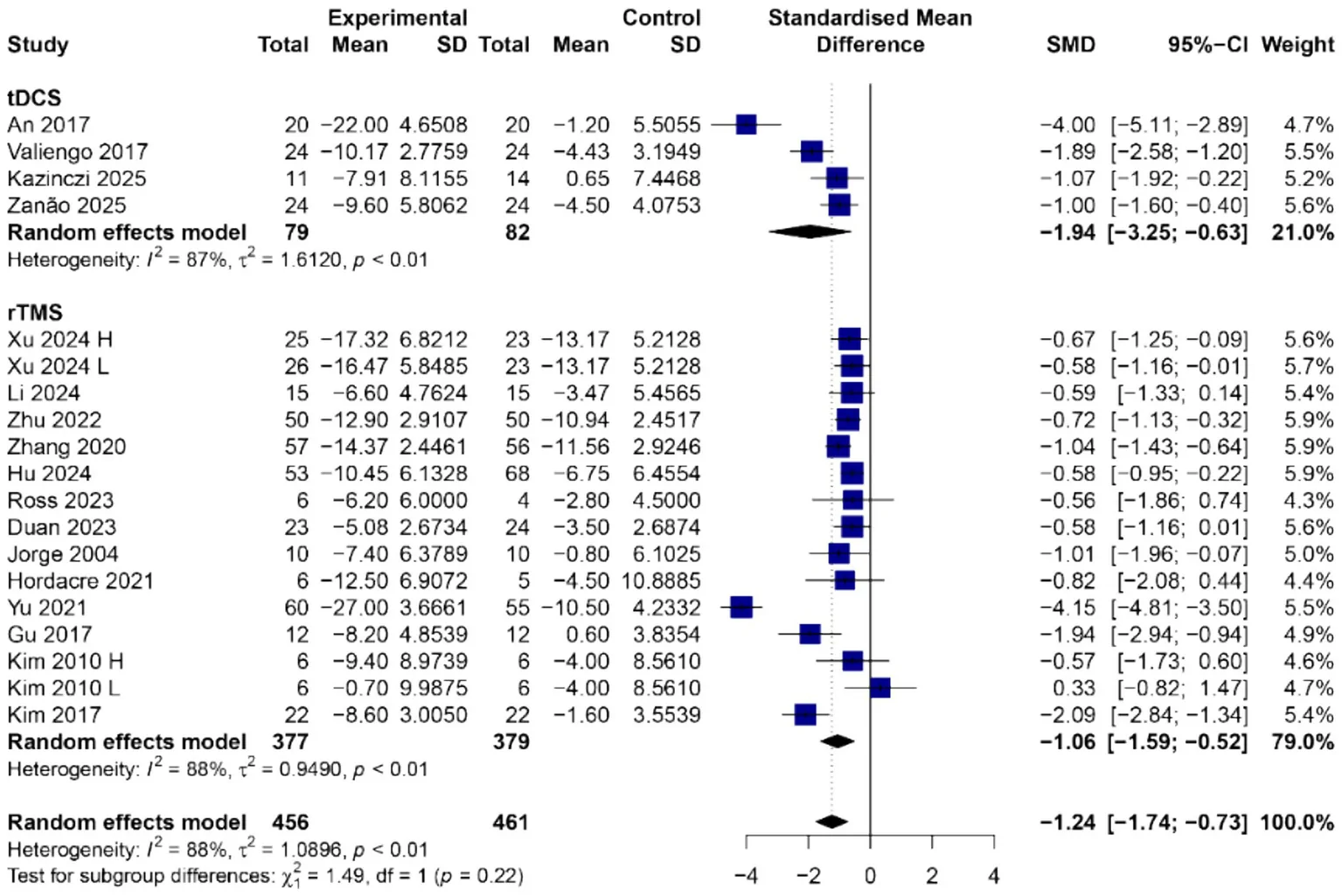
Forest plot of the effects of NIBS on depressive symptoms: subgroup analysis by stimulation modality.

Subgroup analysis by stimulation modality showed significant antidepressant effects for both tDCS and rTMS. The tDCS subgroup included four studies and showed a pooled SMD of −1.94 (95% CI: −3.25 to −0.63), indicating a significant reduction in depressive symptoms. The rTMS subgroup also showed a significant effect (SMD = −1.06, 95% CI: −1.59 to −0.52). Heterogeneity was high in both subgroups (tDCS: *I*^2^ = 87%, τ^2^ = 1.6120, *p* < 0.01; rTMS: *I*^2^ = 88%, τ^2^ = 0.9490, *p* < 0.01). Although the pooled effect appeared numerically larger for tDCS, the between-subgroup difference was not statistically significant (χ^2^ = 1.49, df = 1, *p* = 0.22) (Figure 4).

**Figure 5:**
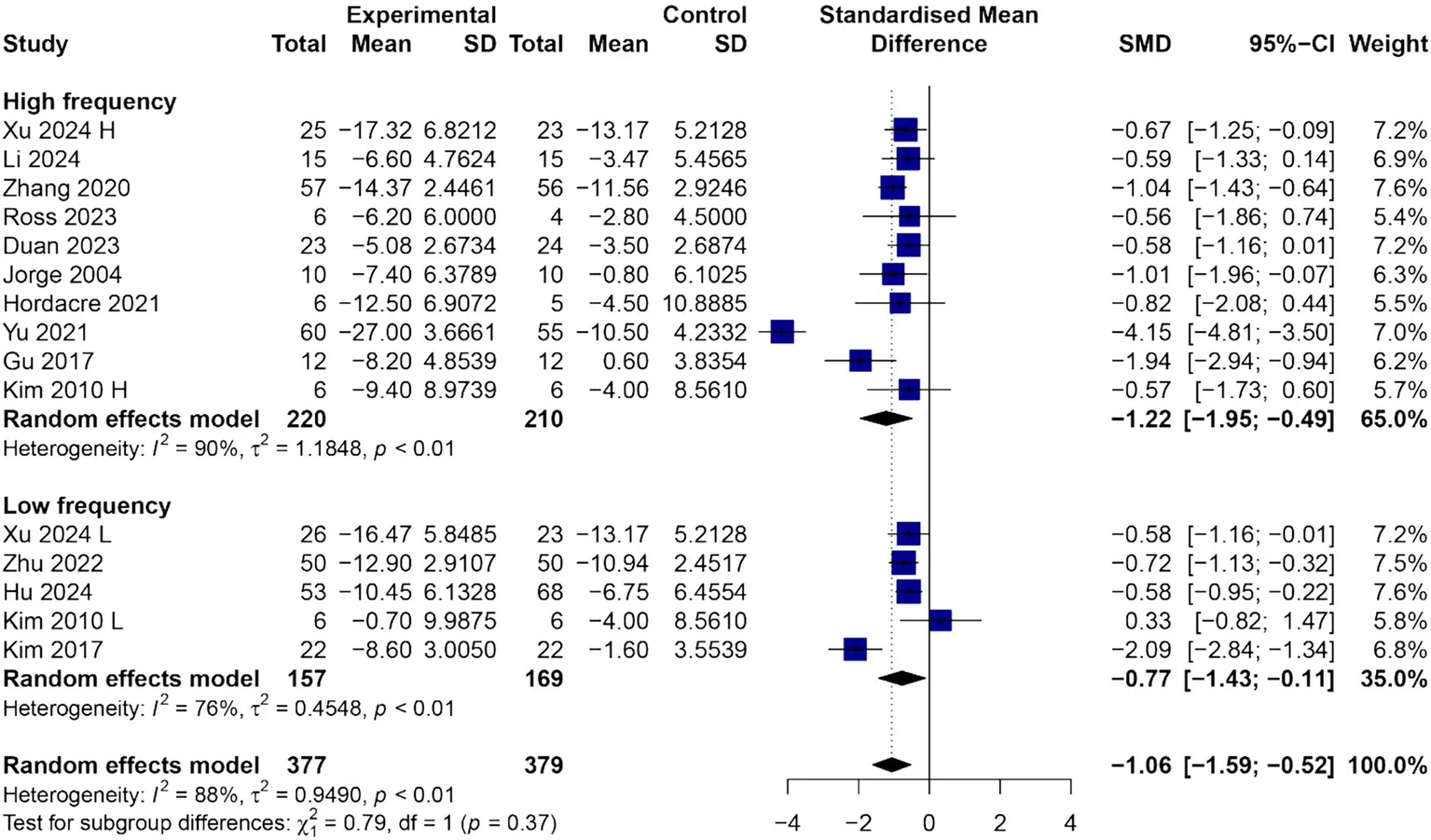
Subgroup analysis of rTMS effects on PSD by stimulation frequency (high vs. low frequency).

A subgroup meta-analysis was conducted to assess whether rTMS frequency influenced antidepressant efficacy. Across rTMS trials, the pooled effect was significant (SMD = −1.06, 95% CI: −1.59 to −0.52, *p* < 0.01). High-frequency protocols (> = 5 Hz) showed a numerically larger effect (SMD = −1.22, 95% CI: −1.95 to −0.49; *I*^2^ = 90%, τ^2^ = 1.1848), whereas low-frequency protocols (<=1 Hz) produced a smaller but significant effect (SMD = −0.77, 95% CI: −1.43 to −0.11; *I*^2^ = 76%, τ^2^ = 0.4548). The between-subgroup comparison was not statistically significant (χ^2^ = 0.79, df = 1, *p* = 0.37) (Figure 5), suggesting that the apparent difference should be interpreted cautiously.

### Therapeutic impact of NIBS on activities of daily living

**Figure 6:**
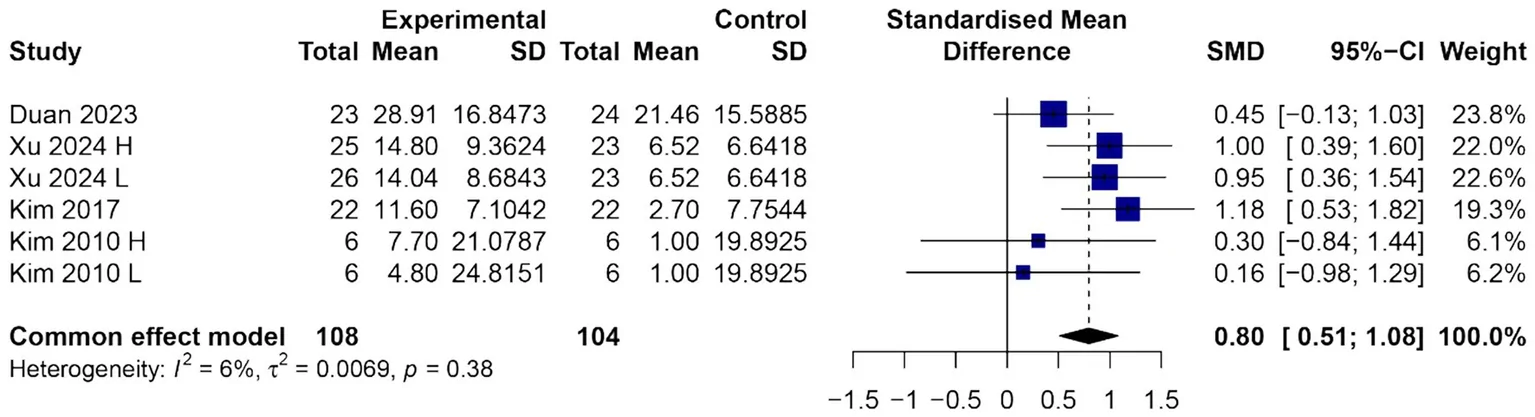
Forest plot of the effects of NIBS on activities of daily living in post-stroke patients.

Six comparisons contributed data to the analysis of activities of daily living (ADL), including 108 participants in the intervention groups and 104 in the control groups. Heterogeneity was low (*I*^2^ = 6%, τ^2^ = 0.0069, *p* = 0.38), and a fixed-effect model was applied. NIBS was associated with better ADL outcomes than control interventions (SMD = 0.80, 95% CI: 0.51–1.08) (Figure 6). Individual estimates generally favored active stimulation, although the magnitude of benefit varied across studies.

#### Therapeutic impact of NIBS on quality of life

**Figure 7:**
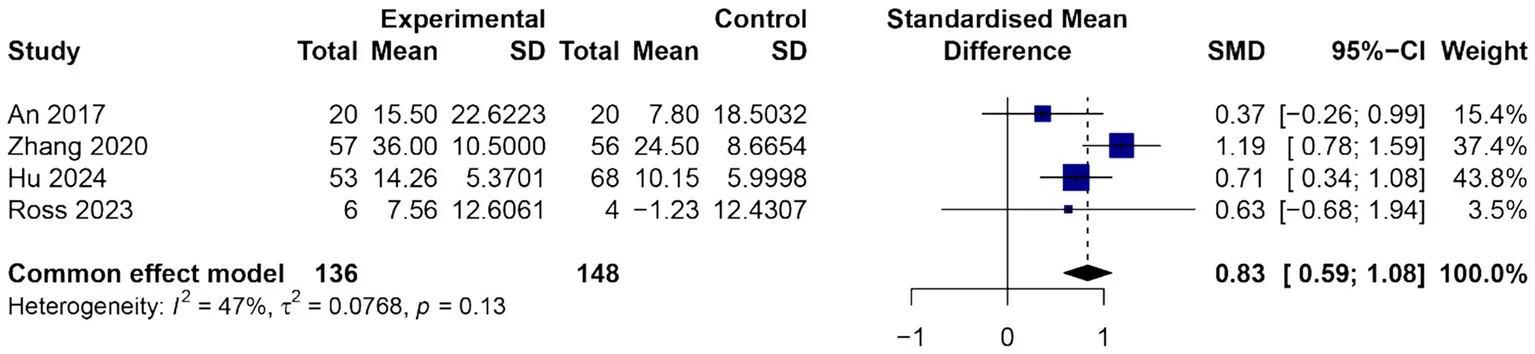
Forest plot of the effects of NIBS on quality of life in individuals with PSD.

Four studies (An et al., 2017; Hu et al., 2024; Ross et al., 2023; Zhang et al., 2020) were included in the meta-analysis of quality of life (QOL), with 136 participants in the intervention groups and 148 in the control groups. A fixed-effect model was applied because heterogeneity was moderate (*I*^2^ = 47%, τ^2^ = 0.0768, *p* = 0.13) (Figure 7). The pooled effect indicated higher QOL scores after NIBS compared with control interventions (SMD = 0.83, 95% CI: 0.59–1.08). Although some individual studies had confidence intervals crossing the null, the overall direction of effect favored active stimulation.

**Figure 8:**
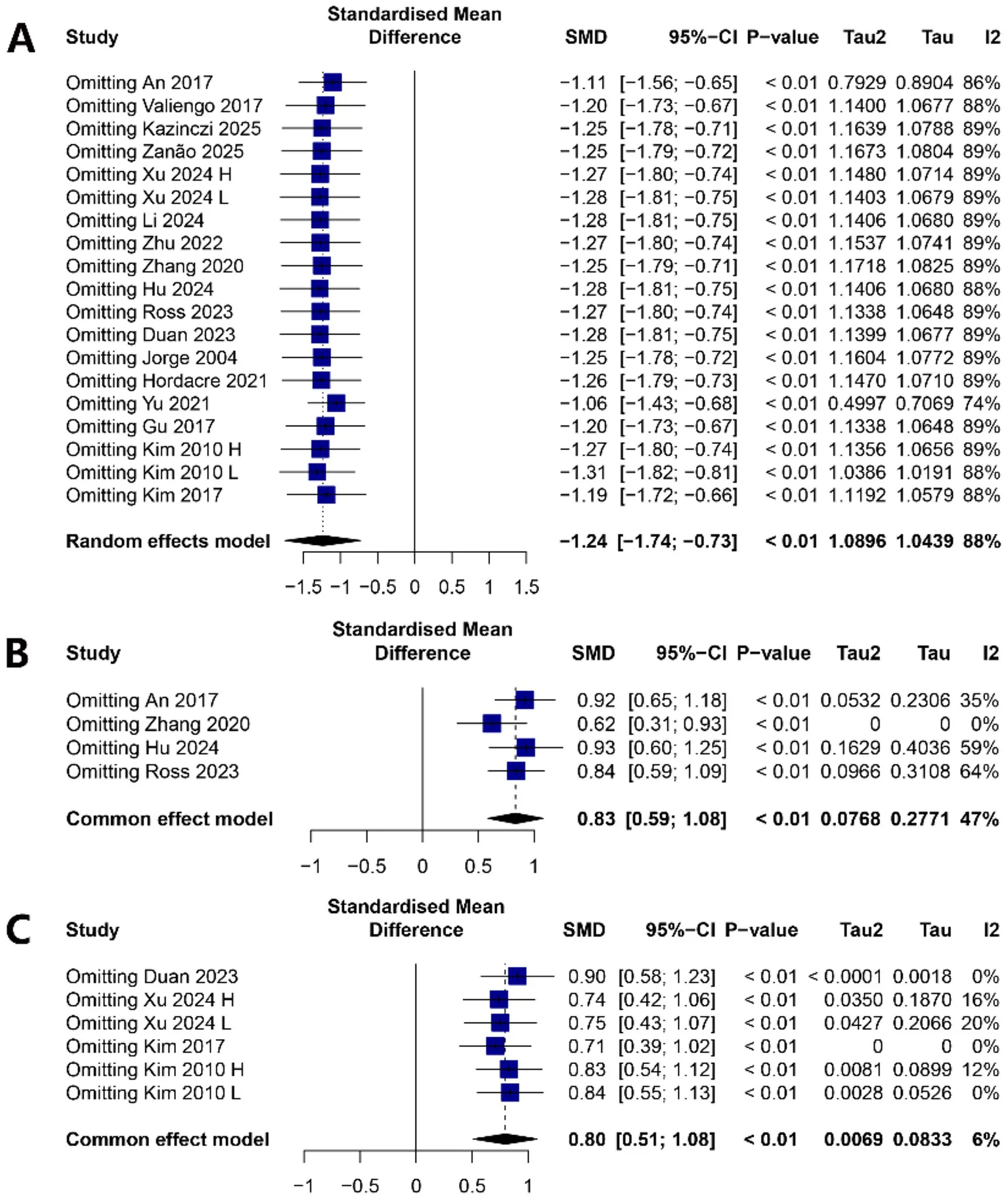
Leave-one-out sensitivity analyses for the effects of NIBS on post-stroke depression (PSD). **(A)** Depression outcomes; **(B)** Quality of life (QOL); **(C)** Activities of daily living (ADL).

Leave-one-out sensitivity analyses were performed for the overall depressive symptom analysis and secondary outcomes (Figure 8). The direction of effect generally remained stable after sequential removal of individual studies, suggesting that the main findings were not driven by any single trial. Nevertheless, the magnitude of the pooled effect varied to some extent, which is consistent with the substantial clinical and methodological heterogeneity observed across the included studies.

**Figure 9:**
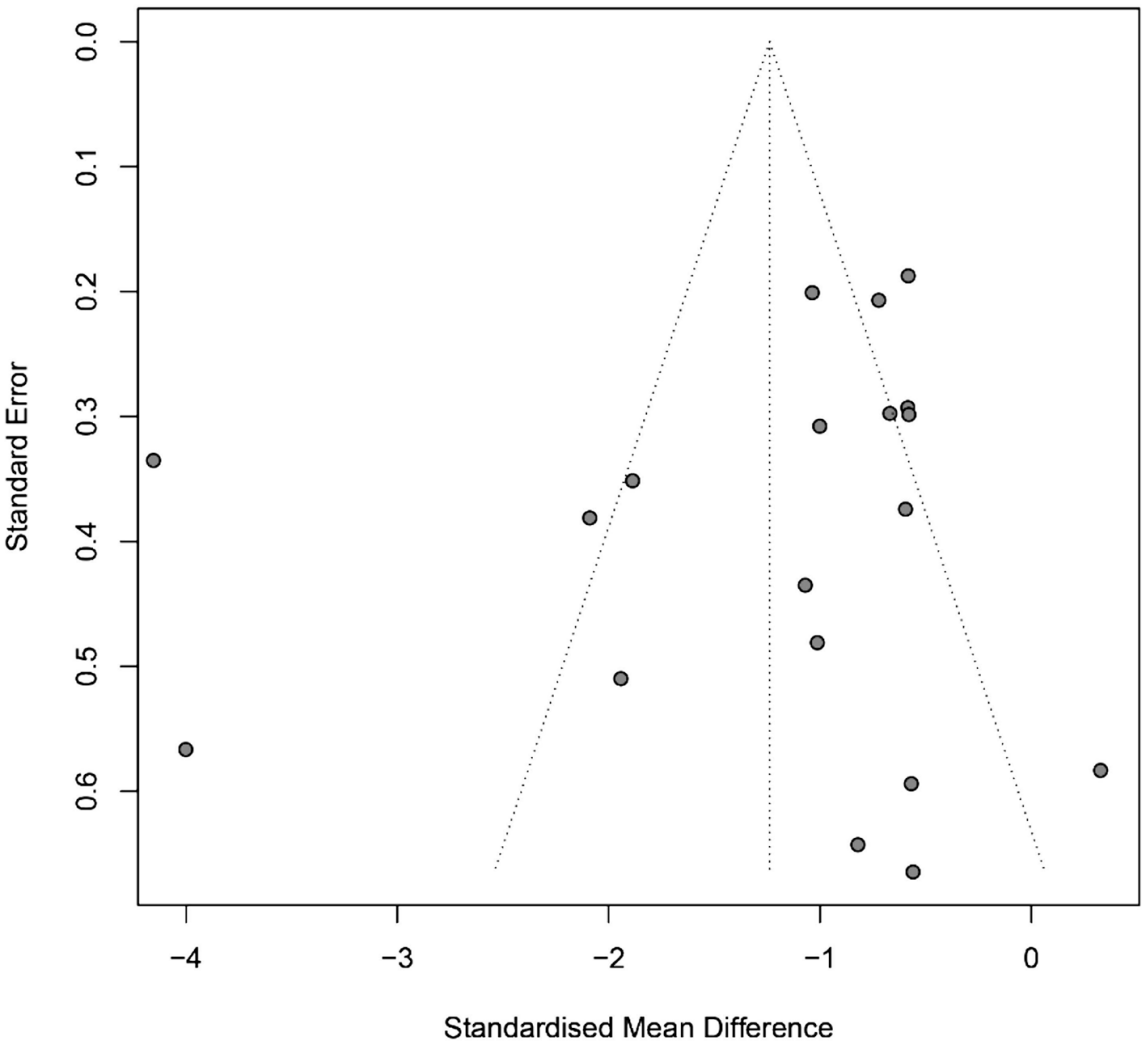
Funnel plot for assessing publication bias.

Potential publication bias was assessed using a funnel plot and Egger’s regression test (Figure 9). Visual inspection did not show marked asymmetry. Egger’s regression test was not statistically significant (*t* = −1.06, df = 17, *p* = 0.3054), suggesting no clear evidence of small-study effects. However, this result should be interpreted cautiously because the number of trials was limited and heterogeneity was substantial.

### Adverse effects

No major adverse events related to NIBS were reported in the included trials. Minor adverse effects, including transient headache, scalp discomfort, itching, or tingling sensations, were occasionally reported, but these events were generally mild and did not require discontinuation of treatment.

## Discussion

This systematic review and meta-analysis synthesized evidence from 17 randomized controlled trials evaluating rTMS and tDCS for PSD. Overall, NIBS was associated with a significant reduction in depressive symptoms compared with control interventions. The pooled effect was moderate to large, but substantial heterogeneity was present, indicating that the magnitude of benefit varied across trials. These findings are broadly consistent with previous reviews suggesting that neuromodulation may reduce depressive symptoms after stroke (Bucur and Papagno, 2018; Shen et al., 2017; Zhang et al., 2024), while also highlighting the need for cautious interpretation because protocols, stimulation sites, comparators, and patient characteristics differed substantially.

The subgroup analysis showed that both tDCS and rTMS were associated with improvements in depressive symptoms, with numerically larger effects observed for tDCS (SMD = −1.94) than for rTMS (SMD = −1.06). However, the between-modality difference was not statistically significant, and both subgroups showed high heterogeneity. Therefore, the results should not be interpreted as definitive evidence that one modality is superior to the other. Similarly, high-frequency rTMS appeared to have a larger effect than low-frequency rTMS, but the subgroup difference was not statistically significant. These findings suggest that stimulation frequency may be clinically relevant, but current data are insufficient to establish an optimal rTMS protocol for PSD.

### Clinical benefits and underlying neurophysiological basis

rTMS and tDCS may improve PSD through partly overlapping but distinct mechanisms. High-frequency rTMS over the left DLPFC may increase cortical excitability and modulate fronto-limbic circuits involved in emotion regulation, whereas low-frequency stimulation may reduce maladaptive activity depending on stimulation site and network state. tDCS may shift resting membrane potentials and influence synaptic plasticity, potentially interacting with rehabilitation-related learning mechanisms and neurotrophic pathways (Fritsch et al., 2010; Lefaucheur et al., 2014). In addition, PSD is often accompanied by impaired motivation, cognitive control, and participation in rehabilitation; therefore, modulation of prefrontal networks may have downstream effects on functional engagement and recovery. These mechanistic explanations remain hypotheses, however, because most included trials did not directly measure neural biomarkers or network changes.

An additional consideration is the state-dependent nature of NIBS. The effects of rTMS and tDCS are not determined only by stimulation parameters, but also by the ongoing functional state of the stimulated networks (Bergmann, 2018; Miniussi et al., 2013; Silvanto et al., 2008). In PSD, treatment responsiveness may therefore be modulated by post-stroke cognitive state, affective state, and neurophysiological state. Cognitive factors such as attention, executive dysfunction, aphasia, and the ability to engage in rehabilitation may influence how patients interact with stimulation and concurrent therapy. Affective factors, including baseline depression severity, anxiety, motivation, and antidepressant use, may alter prefrontal-limbic network responsiveness. Neurophysiological factors, such as lesion location, post-stroke stage, corticospinal excitability, interhemispheric imbalance, and residual network connectivity, may further determine whether DLPFC stimulation restores adaptive activity or produces weaker effects. This state-dependency framework may partly explain the heterogeneity observed across trials, because similar stimulation protocols may have different effects when applied to patients with different cognitive-affective and neural states. Future PSD trials should therefore characterize baseline clinical and neurophysiological states and consider state-stratified or state-guided stimulation approaches.

Home-based tDCS deserves specific consideration because it may reduce the access burden associated with repeated stimulation sessions. Evidence from depressive-disorder populations suggests that home-based tDCS can be feasible and may reduce depressive symptoms, but adherence, supervision, skin-related adverse events, and patient selection remain important concerns (Moshfeghinia et al., 2025). In the PSD context, home-based protocols may be more complex because patients may have cognitive impairment, motor disability, aphasia, or caregiver dependence. Therefore, although home-based tDCS is promising, its use in PSD should be evaluated in dedicated trials with adequate monitoring and safety procedures before routine implementation.

### Broader therapeutic effects, safety profile, and clinical integration

Beyond depressive symptoms, NIBS was associated with improvements in ADL and QOL, with pooled SMDs of 0.80 and 0.83, respectively. These findings may reflect both direct mood-related effects and indirect benefits through greater engagement in rehabilitation. However, the number of studies reporting these secondary outcomes was limited, and the instruments used differed across trials. Therefore, these results should be considered supportive rather than definitive. The safety profile was generally favorable, with no serious stimulation-related adverse events reported and only mild transient discomfort described in several trials.

From a clinical perspective, NIBS may be considered as an adjunctive option within multidisciplinary PSD rehabilitation rather than as a stand-alone replacement for established care. Its potential role may be greatest for patients with persistent depressive symptoms, limited response to pharmacological therapy, or contraindications to antidepressants. Nevertheless, clinical implementation requires standardized stimulation protocols, careful screening for contraindications, monitoring of adverse effects, and integration with psychological, pharmacological, and rehabilitation interventions. Future trials should compare stimulation modalities directly, standardize outcome timing, and evaluate whether improvements in depressive symptoms translate into durable gains in participation and function.

Several limitations should be acknowledged. First, heterogeneity was substantial in the primary depression analysis and in several subgroups, likely reflecting differences in stimulation parameters, treatment duration, comparator conditions, PSD stage, and outcome measures. Second, some trials had small sample sizes or methodological concerns, which may reduce the robustness of pooled estimates. Third, evidence for secondary outcomes and long-term effects remains limited. Fourth, although the review focused on rTMS and tDCS because these modalities had the most relevant randomized evidence in PSD, other NIBS approaches such as intermittent theta-burst stimulation and transcranial alternating current stimulation are emerging and should be considered in future evidence syntheses (Alexander et al., 2019; Wang et al., 2025). Fifth, most included trials did not systematically report baseline cognitive, affective, or neurophysiological profiles, preventing formal evaluation of state-dependent moderators of NIBS response.

## Conclusion

This meta-analysis suggests that rTMS and tDCS may reduce depressive symptoms in patients with PSD and appear generally well tolerated. NIBS may also be associated with improvements in ADL and QOL, but the supporting evidence is less extensive. Because substantial heterogeneity and methodological limitations remain, these findings should be interpreted cautiously. Larger, well-designed randomized trials are needed to define optimal stimulation parameters, identify suitable patient subgroups and state-dependent moderators of response, and determine the durability and clinical significance of treatment effects.

## Data Availability

The raw data supporting the conclusions of this article will be made available by the authors, without undue reservation.
