# Supplementary material for: Efficacy of non-invasive brain stimulation for post-stroke depression: a systematic review and meta-analysis
Source: Front Psychol. 2026 Aug 27;17:1870615. doi: 10.3389/fpsyg.2026.1870615 (PMC13558183; doi:10.3389/fpsyg.2026.1870615)
Supplement: Supplementary file 1 [file Supplementary_file_1.docx]

**Supplementary Table S1. Database-specific search strategy**

**PubMed**

Fields: MeSH and Title/Abstract.

Strategy: (("Stroke"[MeSH] OR stroke[tiab] OR poststroke[tiab] OR post-stroke[tiab]) AND ("Depression"[MeSH] OR depression[tiab] OR depressive[tiab] OR "post-stroke depression"[tiab]) AND ("Transcranial Magnetic Stimulation"[MeSH] OR rTMS[tiab] OR "repetitive transcranial magnetic stimulation"[tiab] OR "Transcranial Direct Current Stimulation"[MeSH] OR tDCS[tiab] OR "transcranial direct current stimulation"[tiab] OR "non-invasive brain stimulation"[tiab] OR NIBS[tiab]))

**Embase**

Fields: Emtree and title/abstract/keyword.

Strategy: ('stroke'/exp OR stroke:ti,ab,kw OR poststroke:ti,ab,kw OR 'post-stroke':ti,ab,kw) AND ('depression'/exp OR depression:ti,ab,kw OR depressive:ti,ab,kw OR 'post-stroke depression':ti,ab,kw) AND ('transcranial magnetic stimulation'/exp OR rtms:ti,ab,kw OR 'repetitive transcranial magnetic stimulation':ti,ab,kw OR 'transcranial direct current stimulation'/exp OR tdcs:ti,ab,kw OR 'non-invasive brain stimulation':ti,ab,kw OR nibs:ti,ab,kw)

**Web of Science**

Fields: Topic.

Strategy: TS=((stroke OR poststroke OR "post-stroke") AND (depression OR depressive OR "post-stroke depression") AND ("non-invasive brain stimulation" OR NIBS OR rTMS OR "repetitive transcranial magnetic stimulation" OR tDCS OR "transcranial direct current stimulation"))

**Cochrane Library**

Fields: Title/Abstract/Keyword.

Strategy: (stroke OR poststroke OR "post-stroke") AND (depression OR depressive OR "post-stroke depression") AND ("non-invasive brain stimulation" OR NIBS OR rTMS OR "repetitive transcranial magnetic stimulation" OR tDCS OR "transcranial direct current stimulation")

**Scopus**

Fields: Title/Abstract/Keyword.

Strategy: TITLE-ABS-KEY((stroke OR poststroke OR "post-stroke") AND (depression OR depressive OR "post-stroke depression") AND ("non-invasive brain stimulation" OR NIBS OR rTMS OR "repetitive transcranial magnetic stimulation" OR tDCS OR "transcranial direct current stimulation"))
